# Complete Genome Sequence of Pseudomonas putida Strain TS312, Harboring an HdtS-Type *N*-Acyl-Homoserine Lactone Synthase, Isolated from a Paper Mill

**DOI:** 10.1128/MRA.00055-20

**Published:** 2020-03-26

**Authors:** Ayaka Hosoe, Toshikazu Suenaga, Takumi Sugi, Taro Iizumi, Naohiro Nagai, Akihiko Terada

**Affiliations:** a Department of Chemical Engineering, Tokyo University of Agriculture and Technology, Koganei, Tokyo, Japan; b Global Innovation Research Institute, Tokyo University of Agriculture and Technology, Fuchu, Tokyo, Japan; c Kurita Analysis Service Co. Ltd., Atsugi, Kanagawa, Japan; d Kurita Global Technology Center, Kurita Water Industries Ltd., Nogi-Machi, Tochigi, Japan; Loyola University Chicago

## Abstract

We report the complete genome sequence of Pseudomonas putida strain TS312, in the class of *Gammaproteobacteria*. The strain, isolated from a paper mill, harbors the *hdtS* gene, encoding *N*-acyl-homoserine lactone synthase. Deciphering the genome contributes to revealing the mechanisms of quorum sensing and associated biofilm formation in engineered systems.

## ANNOUNCEMENT

Cooling water, paper mills, and water reclamation systems are sites where microbial biofilms grow. The formation of a biofilm in these engineered systems affects thermal efficiency (1), product quality (2), and water filtration (3), requiring the mitigation of biofilm formation. In engineered systems in which biofilms are detrimental, antibacterial reagents have been broadly applied. However, care should be taken because the discharged residual reagents may have harmful environmental effects (4). Quorum sensing (QS) is a bacterial cell-cell communication mechanism in which bacterial cells sense others via a signal molecule whose external concentration depends on the cell population density. Exceeding a threshold concentration of a signal compound triggers the expression of specific genes associated with virulence, fluorescence, biofilm formation, or other processes (5). *N*-Acyl-homoserine lactone (AHL)-mediated QS is often used by diverse bacteria belonging to *Gammaproteobacteria*, e.g., Aeromonas hydrophila and Pseudomonas aeruginosa (6).

Pseudomonas putida strain TS312, reported in this study, was isolated from process water (white water) in a Japanese paper mill. Reportedly, P. putida produces AHLs to regulate biofilm formation (7–9). However, the in-depth metabolic potential and AHL-dependent QS mechanisms of P. putida strain TS312 have yet to be deciphered. Understanding the physiological traits of P. putida strain TS312 may provide clues to suppressing biofouling in engineered systems where biofilms are unwanted.

White water was taken from the paper mill, serially diluted, and spread onto an agar medium consisting of 1 g/liter of polypeptide, 1 g/liter of yeast extract, 0.5 g/liter of NaCl, and 15 g/liter of agar. After incubation at 30°C for 7 days, colonies were picked up, followed by identification based on the 16S rRNA gene. A single colony of the isolate, P. putida strain TS312, was suspended, grown aerobically in R2A broth (DAIGO; Fujifilm Holdings Corp., Tokyo, Japan), and subsequently harvested as a cell pellet by centrifugation. Genomic DNA was extracted by a phenol-chloroform extraction method (10) and subsequently purified with cetyltrimethylammonium bromide. RNA contaminating the genomic DNA was degraded with RNase A (TaKaRa Bio, Inc., Shiga, Japan). The DNA library was prepared as reported previously (11, 12). In brief, the library was prepared using a 1D ligation sequencing kit (SQK-LSK-109; Oxford Nanopore Technologies Ltd., Oxford, UK), without a fragmentation procedure, and sequenced on the MinION Mk1B system using an R.10 flow cell (FLO-MIN110; Oxford Nanopore Technologies). The sequence data were base called by Guppy (version 3.3.2) using the high-accuracy mode. The sequences produced included 397,900 reads, consisting of 5.73 Gbp in total, with an *N*_50_ value of 26.21 kbp. The sequence quality was confirmed by NanoPlot (13), and the adaptor sequences, low-quality reads (score of <Q10), and short reads (<1,000 bp) were removed using Porechop as a tool for trimming adapters. The quality control-passed sequences totaled 806 Mbp and were used for further analysis. The acquired sequences were assembled using Canu (version 1.8) (14), and only 1 suggested circular contig was generated, with 141-fold coverage of the genome. The consensus sequence was polished by medaka (version 0.10.1), and the completeness, as assessed by BUSCO (version 1) (15), was 100%. The parameters used for the bioinformatics software were the default values unless otherwise noted. The complete genome sequence was annotated using the DDBJ Fast Annotation and Submission Tool (16). The genome consists of 5,681,150 bp in 1 contig, with a G+C content of 66.2%, containing 5,110 protein-coding DNA sequences, 22 rRNAs, and 75 tRNAs.

Based on a BLAST search, P. putida strain TS312 harbors typical functional genes encoding AHL acylase, i.e., PvdQ and QuiP (17, 18). Strain TS312 also contains a third type of AHL synthase, HdtS, a member of the lysophosphatidic acid acyl transferase family possessed by Pseudomonas fluorescens (19). The HtdS family performs both acylation of lysophosphatidic acid and synthesis of AHLs; however, the implication of HdtS in the synthesis of AHLs has not been fully clarified (20). Further research to elucidate the roles of HdtS in biofilm formation by P. putida strain TS312 could lead to the development of a strategy to combat unwanted biofilm formation.

### Data availability.

The complete genome sequence of P. putida strain TS312 has been deposited in GenBank under accession number AP022324 and BioProject number PRJDB9166. The raw data are available in the DDBJ Sequence Read Archive (DRA) under accession number DRA009539. The data addressed in this paper represent the first version.
